# Predictors of Symptom Reduction and Remission Among People with Anxiety: Secondary Analyses from a Randomized Controlled Trial

**DOI:** 10.1007/s11126-024-10081-y

**Published:** 2024-07-18

**Authors:** Marte Ustrup, Thomas Christensen, Nadja Kehler Curth, Kimmie Heine, Anders Bo Bojesen, Lene Falgaard Eplov

**Affiliations:** Copenhagen Research Unit for Recovery, Mental Health Center Amager, Mental Health Services in the Capital Region of Denmark, Hans Bogbinders Allé 3, 2300 Copenhagen, Denmark

**Keywords:** Anxiety, Anxiety disorders, Collaborative Care, Predictors, Prognosis, Remission

## Abstract

Despite the substantial disease burden of anxiety disorders, only limited or conflicting data on prognostic factors is available. Most studies include patients in the secondary healthcare sector thus, the generalizability of findings is limited. The present study examines predictors of symptom reduction and remission in patients with anxiety disorders in a primary care setting. 214 patients with anxiety disorders, recruited as part of the Collabri Flex trial, were included in secondary analyses. Data on potential predictors of anxiety symptoms at 6-month follow-up was collected at baseline, including patient characteristics related to demography, illness, comorbidity, functional level, life quality, and self-efficacy. The outcomes were symptom reduction and remission. Univariate and multivariate linear and logistic regression analyses were conducted to assess the associations between predictor variables and the outcome, and machine-learning methods were also applied. In multiple linear regression analysis, anxiety severity at baseline (*β* = -6.05, 95% CI = -7.54,-4.56, p < 0.001) and general psychological problems and symptoms of psychopathology (SCL-90-R score) (*β* = 2.19, 95% CI = 0.24,4.14, p = 0.028) were significantly associated with symptom change at 6 months. Moreover, self-efficacy was associated with the outcome, however no longer significant in the multiple regression model. In multiple logistic regression analysis, anxiety severity at baseline (OR = 0.54, 95% CI = -1.13,-0.12, p = 0.018) was significantly associated with remission at 6 months. There was no predictive performance of the machine-learning models. Our study contributes with information that could be valuable knowledge for managing anxiety disorders in primary care.

**Supplementary Information:**

The online version contains supplementary material available at 10.1007/s11126-024-10081-y.

## Introduction

Worldwide, mental disorders are among the top ten leading causes of disease burden, and anxiety disorders constitute the largest group of mental disorders in most high-income countries [1, 2]. Anxiety disorders accounted for an estimated 301.4 million cases in 2019, corresponding to an increase of 55% between 1990 and 2019 [1]. It is estimated that mental disorders cost the world economy approximately 2.5 trillion US Dollars per year in disability and lost productivity [3]. Despite their public health and economic significance, the majority of anxiety disorders remain undetected and untreated by healthcare systems, especially in primary care settings [2]. If untreated, these disorders are characterized by fluctuating symptom levels, a long time to remission, and high rates of recurrence [2, 4, 5]. Prognosis varies across disorders; most studies find favorable remission rates for specific phobias, gradually declining for generalized anxiety disorder and social anxiety disorder to low remission rates for panic disorder and for those with multiple anxiety disorders [4, 6–8]. Several studies have investigated the relationship between specific predictors and either remission of anxiety or poor remission/recurrence of anxiety. However, study design and sample sizes vary to a great extent between studies, many only cover a few variables, and are limited to one or few anxiety disorders [9]. In addition, most studies are conducted in psychiatric settings [6, 10–12]. Consequently, results can be inconclusive, difficult to compare, and mainly refer to severe anxiety disorders. With this in mind, poor remission has been predicted by risk factors within several domains, such as demographic characteristics (e.g. being single, having an immigrant background, experiencing limited social support), financial characteristics (e.g. low socioeconomic status, unemployment, receiving disability income), health-related characteristics (e.g. comorbid depression, comorbid personality disorder, antidepressant medication use at baseline), and psychological characteristics (e.g. low extraversion, longer duration of avoidance behavior, neuroticism) [6, 10, 13–16]. However, for some of these factors, findings from the literature are contradictory, e.g. for anxiety severity at baseline and comorbidities [13, 14, 17–19]. Studies on the association between poor anxiety remission and gender or age also yield conflicting results [6]. In contrast, symptom reduction and remission are predicted by protective factors, such as higher socioeconomic status, employment, being Caucasian, experiencing a great amount of social support, less severe anxiety at baseline, less comorbidity, positive mental health, and taking sleep medication [13–15, 20, 21]. Furthermore, protective factors include high self-reported self-efficacy and outcome-expectancy [13, 22, 23].

As noted by the authors, limitations exist in the literature on predictors of anxiety symptom reduction and remission, hence limiting their generalizability. The Collabri Flex study offers a possibility to examine predictors in different anxiety disorders identified in a primary care setting. Two Collabri Flex randomized controlled trials (RCTs) were initiated in 2018 in the Capital Region of Denmark, investigating the effects of collaborative care versus consultation liaison for anxiety disorders and depression, respectively [24]. The studies demonstrated that collaborative care was an effective model for improving outcomes at 6 months for persons with depression or anxiety disorders [25]. In addition to demonstrating the effectiveness of collaborative care, the Collabri Flex trials provided important secondary data for analyzing specific predictors of anxiety and depression symptom reduction and remission.

The aim of the present study is to examine predictors of anxiety symptom reduction and remission after 6 months in patients identified in a primary care setting with social anxiety disorder, panic disorder/agoraphobia, obsessive–compulsive disorder, generalized anxiety disorder, and post-traumatic stress disorder.

## Methods

The present study was designed as a prospective cohort study, including secondary analyses of data obtained from the Collabri Flex trial on anxiety disorders. The methodology of the two Collabri Flex trials has been described in detail elsewhere and will only be briefly summarized in the following section [24].

### Overall Design of the Original RCT

The Collabri Flex RCTs were investigator-initiated randomized parallel superiority trials for anxiety disorders and depression, respectively, investigating the effects of collaborative care versus consultation liaison.

Participants were recruited through general practitioners, thus representing the primary care sector. For the anxiety trial, subjects were eligible if they had an ICD-10 diagnosis of social anxiety disorder, panic disorder/agoraphobia, obsessive–compulsive disorder, generalized anxiety disorder, or post-traumatic stress disorder. Furthermore, inclusion criteria included the ability to speak Danish, age 18 years or older, and provision of written consent to participate [24].

In total, 1,010 participants were assessed for eligibility, and 309 did not meet the inclusion criteria or were excluded according to the exclusion criteria [24]. This resulted in the recruitment of 303 participants with anxiety disorders and 398 participants with depression for the two RCTs, respectively [25].

### Design of the Predictor Analyses

The present study examined potential predictors of anxiety symptom reduction and remission after 6 months in patients with anxiety disorders.

#### Outcome

The outcome of interest was self-reported anxiety symptoms, measured by the Beck Anxiety Inventory (BAI) at 6-month follow-up [26]. BAI is a 21-item questionnaire assessing the severity of anxiety [26]. Each of the 21 items is scored on a scale value of 0 (not at all) to 3 (severely) and summed to a total score of maximum 63. Scores between 0–9 indicate a minimal or no level of anxiety, scores between 10–18 indicate mild to moderate anxiety, scores between 19–29 indicate moderate to severe anxiety, and scores between 30–63 indicate severe anxiety [27]. In this study, two different definitions of the 6-month outcome ‘anxiety symptoms’ (BAI score) were used: (1) Anxiety symptom reduction, measured as a continuous variable, referred to experiencing symptom improvement, and (2) Anxiety remission, measured as a binary categorical variable, indicated that symptoms had been mostly alleviated. Clinically relevant symptom change was defined as a minimum 4-point difference in anxiety symptoms between baseline and 6-month follow-up. Remission was defined as a cut-off value of BAI score < 10 at 6-month follow-up.

#### Predictor Variables

##### Demographic Variables

Data was collected on demographic variables, including sex, age, and marital status. Educational level was assessed as either Lower Secondary, Upper Secondary, Vocational, Bachelors’ degree, Masters’ degree, or Ph.D. Employment status was assessed as either employed, under education, or unemployed. These variables were collected through Danish national registers.

##### Primary Illness Variables

Types of anxiety disorders included social anxiety disorder, panic disorder/agoraphobia, obsessive–compulsive disorder, generalized anxiety disorder, or post-traumatic stress disorder. After referral of a participant to the trial from general practitioners, a care manager assessed the type of anxiety disorder, using the MINI International Neuropsychiatric Interview [28] supplemented with ICD-10 specific questions.

Through questionnaires administered at baseline, self-reported measures were obtained on anxiety severity at baseline (BAI) and on previous psychological or medical treatment for anxiety or depression. The latter was not restricted to a predefined period, but covered any treatment through lifetime, and was included to serve as a proxy for illness severity.

##### Comorbidity Variables

Comorbid depression was measured by the Beck Depression Inventory (BDI-II), which is a 21-item questionnaire assessing the severity of depression [29]. Each of the 21 items is scored on a scale value of 0 (not at all) to 3 (severely) and summed to a total score with four cut-off scores indicating the depression severity. Scores between 0–13 indicate a minimal or no level of depression, scores between 14–19 indicate mild depression, scores between 20–28 indicate moderate depression, and scores between 29–63 indicate severe depression [30].

General psychological problems and symptoms of psychopathology were measured by the Symptom Checklist (SCL-90-R), which is a 90-item questionnaire [31, 32]. In the present study, we report the total item-score of the SCL-90-R, which can be converted to the Global Severity Index (GSI) by dividing the total item-score by 90.

Screening for personality disorder was based on the Structured Assessment of Personality Abbreviated Scale (SAPAS) [33]. The SAPAS is an 8-item questionnaire. Each item is answered with yes or no and it produces a score that ranges from 0 to 8.

##### Functional Level Variables

Disability and functional impairment was measured by the Sheehan Disability Scale (SDS) [34, 35]. The SDS is a short measure of disability and functional impairment in three interrelated domains; work/school, social, and family life, scored from 0 to 10 and summed to a total score ranging from 0 (unimpaired) to 30 (highly impaired).

##### Life Quality Variables

Well-being was measured by the WHO Well-Being Index (WHO-5) [36, 37], which is a 5-item questionnaire, where items are summed and multiplied by four, giving a score between 0 and 100 (perfect functioning). A score ≤ 50 indicates poor well-being.

Health-related quality of life was measured by the European Quality of Life Five Dimensions Questionnaire (EQ-5D-3L) [38]. The EQ-5D-3L assesses health status in terms of five dimensions of health, with three response levels of severity (1 = no problems, 2 = some problems, 3 = extreme problems) for each dimension. In the present study, we convert and report the EQ-5D-3L health states into a single index value, where the score 1 indicates full health and 0 corresponds to death.

##### Self-Efficacy Variables

Self-efficacy was measured by the ‘Personal Control subscale’ from the Illness Perception Questionnaire Revised (IPQ-R) [39] and two subscales (‘Self-efficacy to obtain help from community, family, friends scale’ and ‘Self-efficacy to control/manage depression scale’) from the Self-Efficacy to Manage Chronic Disease (SEMCD) scale [40]. The IPQ-R is an 84-item questionnaire measuring an individual’s beliefs and feelings about their illness. It is divided into three sections; Identity subscale, Causal subscale, and a third section, which contains 7 subscales, including the Personal Control subscale. The scores can range from 6 to 30. The SEMCD Scale is a 33-item scale, where the patient rates his/her confidence related to 10 tasks (subscales) on a scale ranging from 1 (not at all confident) to 10 (totally confident). The subscale ‘Self-efficacy to obtain help from community, family, friends’ has 4 items, and the subscale ‘Self-efficacy to control/manage depression’ has 6 items.

### Statistical Analyses

All original analyses were based on the intention to treat principles. Missing data on baseline predictors were handled by multiple imputations, and predictors with missing data had imputed values using the mean of 5 imputation sets. Imputation was carried out using predictive mean matching. Observations with missing endpoint data on BAI were excluded.

Overall, we applied the method of triangulation, by combining multiple methods of analyzing data to cross-check the credibility and validity of our findings.

#### Descriptive Analyses

Baseline characteristics were presented with count (n) and percentages for categorical variables and with mean and standard deviations (SD) for continuous variables.

#### Analyses of Potential Predictors of Anxiety Symptom Reduction

Initially, data was summarized in a correlation matrix to show correlation coefficients between the variables, using Pearson correlation coefficient and Spearman’s rank correlation coefficient. Univariate linear regression analysis was conducted to assess the unadjusted associations between possible baseline predictor variables and anxiety symptom reduction (measured as a continuous variable) at 6-month follow-up. All predictor variables associated with the outcome measure with a p-value < 0.1 were included in multiple linear regression analysis.

#### Analyses of Potential Predictors of Anxiety Remission

Univariate logistic regression analysis was conducted to assess the unadjusted associations between possible baseline predictor variables and anxiety remission (measured as a binary categorical variable) at 6-month follow-up. All predictor variables associated with the outcome measure with a p-value < 0.1 were included in multiple logistic regression analysis.

#### Machine-Learning Methods as Predictive Models

The association between baseline variables and the outcome was additionally tested using two machine-learning methods: random forests [41] and XGBoost [42]. The outcome was the log of the ratio to the baseline measure of BAI of the 6-month measurement to eliminate the impact of the baseline symptom severity. No tuning was done. The number of trees in the random forest was 500. The number of boosting iterations was set at 25. The two machine-learning algorithms are widely used, and computationally efficient, and both are suited for identifying non-linear predictors. Separate models were fitted to five training sets and predictions were applied to the five non-overlapping held-out test sets in a cross-validation regime to avoid overfitting impacting predictions. Correlations between predictions and actual outcomes were used to assess model fit.

#### Control for Treatment Moderating Effects

Since all participants in the RCT received an intervention (collaborative care versus consultation liaison), we subsequently controlled for treatment moderating effects of the observed associations, using: (a) Linear and logistic regression analyses with interaction terms, and (b) Causal forest machine-learning model.

##### Linear and Logistic Regression Analyses with Interaction Terms

To control for treatment moderating effects, we estimated this effect directly by adding interaction terms in the linear and logistic regression analyses. We added interaction terms between significant predictor variables, i.e. variables that were significantly associated with the outcome measure with a p-value < 0.1, and the treatment condition variable.

##### Causal Forest Machine-Learning Model

To explore complex and/or non-linear treatment moderating effects, a causal forest machine-learning model was applied, i.e. the model was used to identify individual patients with a high chance of benefitting from collaborative care.

The causal forest procedure estimated the unobserved individual treatment effect. This method is similar to regular random forests, but instead of optimizing trees to explain differences on an observed outcome, each node in the tree is optimized for maximizing the distance on an outcome for the treated versus comparison group in that node [43]. Again, separate models were fitted to five training sets and predictions were applied to the five non-overlapping held-out test sets in a cross-validation regime to avoid overfitting impacting predictions.

Data were analyzed using R statistical software, version 4.0.

## Results

The present study included 214 participants with anxiety disorders and non-missing endpoint data. In Table 1, characteristics related to baseline demography, illness, comorbidity, functional level, life quality, and self-efficacy are presented for the total population and separately for those who experienced remission (n = 58, 27.1%) versus no remission (n = 156, 72.9%) at 6-month follow-up. Of the total population, 29.5% were diagnosed with generalized anxiety disorder, 47.4% with panic disorder/agoraphobia, and 23.1% with social anxiety disorder/obsessive–compulsive disorder. Due to few participants with obsessive–compulsive disorder, this diagnosis was combined with social anxiety disorder. No participants presented with a post-traumatic stress disorder diagnosis. Seventy percent were female, and the average age was 37 years at the time of inclusion. The mean anxiety score (mean = 25.45) corresponds to a moderate to severe level of anxiety. In addition, comorbid depression was common with a mean depression score (mean = 21.76) corresponding to a moderate level of depression.

**Table 1:** Baseline characteristics of participants with anxiety disorders in the Collabri Flex trial

	**No remission**^**a**^**,** ***N* = 156**		**Remission**^**b**^**,** ***N* = 58**		**Total sample** ***N* = 214**	
**Demographic variables**	**Mean**	**SD**	**Mean**	**SD**	**Mean**	**SD**
**Age**	36.33	14.40	37.48	15.37	36.64	
	***N***	**(%)**	***N***	**(%)**	***N***	**(%)**
**Sex**						
Female	110	70.5	40	69.0	150	70.1
Male	46	29.5	18	31.0	64	29.9
**Marital status**						
Not married	116	74.4	43	74.1	159	74.3
Married	40	25.6	15	25.9	55	25.7
**Educational level**						
Lower Secondary	26	16.7	6	10.3	32	15.0
Upper Secondary	69	44.2	27	46.6	96	44.9
Vocational or Bachelors’ degree	35	22.4	10	17.2	45	21.0
Masters’ degree or Ph.D	26	16.7	15	25.9	41	19.2
**Employment status**						
Employed	62	39.7	30	51.7	92	43.0
Under education	42	26.9	11	19	53	24.8
Unemployed	52	33.3	17	29.3	69	32.2
**Primary illness variables**						
**Diagnosis (type of anxiety disorder)**						
Generalized anxiety disorder	46	29.5	17	29.3	63	29.4
Panic disorder/agoraphobia	74	47.4	31	53.4	105	49.1
Social anxiety disorder/obsessive–compulsive disorder	36	23.1	10	17.2	46	21.5
**Previous psychological/medical treatment**						
No previous treatment	82	52.6	29	50	111	51.9
Previous treatment	74	47.4	29	50	103	48.1
	**Mean**	**SD**	**Mean**	**SD**	**Mean**	**SD**
**Anxiety severity (BAI**^**c**^**)**	27.01	8.41	21.24	8.15	25.45	8.71
**Comorbidity variables**						
**Comorbid depression severity (BDI-II**^**d**^**)**	23.33	9.23	17.53	9.29	21.76	9.57
**General psychological problems and symptoms of psychopathology (SCL-90-R**^**e**^**)**	118.28	45.71	89.09	39.43	110.37	45.97
	***N***	**(%)**	***N***	**(%)**	***N***	**(%)**
**Personality disorder (SAPAS**^**f**^** > 2)**	61	39.1	18	31.0	79	36.9
**Functional level variables**	**Mean**	**SD**	**Mean**	**SD**	**Mean**	**SD**
**Disability and functional impairment (SDS**^**g**^**)**	16.83	6.86	13.91	7.53	16.04	7.15
**Life quality variables**						
**Well-being (WHO5**^**h**^**)**	35.56	17.36	40.76	20.18	36.97	18.27
**Health-related quality of life (EQ-5D-3L**^**i**^**)**	0.66	0.18	0.74	0.12	0.68	0.17
**Self-efficacy variables**						
**Self-efficacy (personal control) (IPQ-R**^**j**^**)**	20.86	3.90	20.93	4.63	20.88	4.10
**Self-efficacy (obtain help) (SEMCD**^**k**^**)**	6.20	2.01	7.03	1.77	6.42	1.98
**Self-efficacy (control/manage symptoms) (SEMCD**^**k**^**)**	5.54	1.84	6.30	1.78	5.75	
**Intervention/treatment condition**	***N***	**(%)**	***N***	**(%)**	***N***	**(%)**
Collabri Flex (CC)	82	52.6	37	63.8	119	55.6
Consultation liaison (CL)	74	47.4	21	36.2	95	44.4

^a^BAI < 10

^b^BAI > 10

^c^BAI: Beck Anxiety Inventory

^d^BDI-II: Beck Depression Inventory

^e^SCL-90-R: Symptom Checklist

^f^SAPAS: Structured Assessment of Personality Abbreviated Scale

^g^SDS: Sheehan Disability Scale

^h^WHO5: WHO Well-Being Index

^i^EQ-5D-3L: European Quality of Life

^j^IPQ-R: Illness Perception Questionnaire Revised

^k^SEMCD: Self-Efficacy to Manage Chronic Disease Scales

### Factors Predicting Anxiety Symptom Reduction

The full correlation matrix is shown in Table S1 (Online Resource). Table 2 presents the results from univariate and multiple linear regression analyses of variables predicting anxiety symptom change. In the univariate analysis, the only *primary illness variable* that was associated with anxiety symptom reduction, measured by change in BAI, at 6 months was ‘Anxiety severity’ at baseline. Thus, a higher anxiety severity at baseline, measured by BAI, was associated with a higher symptom reduction at 6 months (*β* = -4.63, 95% CI = -5.78,-3.47, p < 0.001), i.e. one extra point in baseline BAI score increased the reduction in 6-month BAI score by 4.63 points. Out of the *comorbidity variables*, two were associated with lower anxiety symptom reduction at 6 months: (a) A higher ‘Depression severity’ at baseline, measured by BDI-II, was associated with a lower reduction in anxiety symptoms at 6 months (*β* = 1.40, 95% CI = 0.10,2.71, p = 0.037), and (b) A higher level of ‘General psychological problems and symptoms of psychopathology’ at baseline, measured by SCL-90-R, was associated with a lower reduction in anxiety symptoms at 6 months (*β* = 2.42, 95% CI = 0.97,3.88, p = 0.001). Finally, the only *self-efficacy variable* that was associated with anxiety symptom reduction at 6 months was the self-efficacy measure ‘Self-efficacy to obtain help from community, family, friends’ from the SEMCD scale. Thus, a higher self-efficacy (to obtain help) at baseline was associated with a higher symptom reduction at 6 months (*β* = -1.30, 95% CI = -2.44,-0.17, p = 0.025).

**Table 2:** Predictors of anxiety symptom change at 6-month follow-up, univariate and multivariate linear regression

	**Univariate**			**Multivariate**		
	***β***	**95% CI**	**p-value**	***β***	**95% CI**	**p-value**
**Demographic variables**						
**Age**	0.04	-1.07,1.15	0.947			
**Sex**						
Male	0.93	-1.63,3.48	0.479			
Female	1					
**Marital status**						
Married	-1.07	-3.67,1.54	0.422			
Not married	1					
**Educational level**						
Lower Secondary	1					
Upper Secondary	-2.51	-5.91,0.89	0.150			
Vocational or Bachelors’ degree	-1.56	-5.41,2.29	0.428			
Masters’ degree or Ph.D	-2.65	-6.58,1.27	0.187			
**Employment status**						
Employed	1					
Under education	0.74	-2.15,3.64	0.616			
Unemployed	1.06	-1.60,3.71	0.436			
**Primary illness variables**						
**Diagnosis (type of anxiety disorder)**						
Generalized anxiety disorder	1					
Panic disorder/ agoraphobia	-1.39	-4.07,1.29	0.312			
Social anxiety disorder/obsessive–compulsive disorder	1.38	-1.86,4.63	0.404			
**Previous psychological/medical treatment**						
No previous treatment	1					
Previous treatment	1.57	-0.70,3.84	0.178			
**Anxiety severity (BAI**^**a**^**)**	-4.63	-5.78,-3.47	**< 0.001**	-6.05	-7.54,-4.56	**< 0.001**
**Comorbidity variables**						
**Comorbid depression severity (BDI-II**^**b**^**)**	1.40	0.10,2.71	**0.037**	-0.11	-1.82,1.59	0.896
**General psychological problems and symptoms of psychopathology (SCL-90-R**^**c**^**)**	2.42	0.97,3.88	**0.001**	2.19	0.24,4.14	**0.028**
**Personality disorder (SAPAS**^**d**^** > 2)**	1.35	-1.00,3.71	0.261			
**Functional level variables**						
**Disability and functional impairment (SDS**^**e**^**)**	0.42	-0.88,1.72	0.526			
**Life quality variables**						
**Well-being (WHO5**^**f**^**)**	0.36	-0.96,1.69	0.592			
**Health-related quality of life (EQ-5D-3L**^**g**^**)**	0.30	-0.97,1.56	0.649			
**Self-efficacy variables**						
**Self-efficacy (personal control) (IPQ-R**^**h**^**)**	-0.08	-1.22,1.07	0.893			
**Self-efficacy (obtain help) (SEMCD**^**i**^**)**	-1.30	-2.44,-0.17	**0.025**	-0.77	-1.97,0.43	0.209
**Self-efficacy (control/ manage symptoms) (SEMCD**^**i**^**)**	-0.84	-2.01,0.33	0.159			
**Intervention/treatment condition**						
Collabri Flex (CC)	-2.69	-4.96,-0.43	**0.021**	-1.41	-2.52,-0.30	**0.014**
Consultation liaison (CL)	1					

^a^BAI: Beck Anxiety Inventory

^b^BDI-II: Beck Depression Inventory

^c^SCL-90-R: Symptom Checklist

^d^SAPAS: Structured Assessment of Personality Abbreviated Scale

^e^SDS: Sheehan Disability Scale

^f^WHO5: WHO Well-Being Index

^g^EQ-5D-3L: European Quality of Life

^h^IPQ-R: Illness Perception Questionnaire Revised

^i^SEMCD: Self-Efficacy to Manage Chronic Disease Scales

In the multiple linear regression analysis, only two variables remained significantly associated with anxiety symptom change at 6 months: (a) A higher ‘Anxiety severity’ at baseline was associated with a higher symptom reduction at 6 months (*β* = -6.05, 95% CI = -7.54,-4.56, p < 0.001), and (b) A higher level of ‘General psychological problems and symptoms of psychopathology’ at baseline was associated with a lower reduction in anxiety symptoms at 6 months (*β* = 2.19, 95% CI = 0.24,4.14, p = 0.028).

No association was found between the *demographic variables* representing socioeconomic status (i.e. marital status, educational level, and employment status), the *primary illness variable* ‘Diagnosis’ (type of anxiety disorder), or the *comorbidity variable* ‘Personality disorder’, and the outcome. Furthermore, the remaining two *self-efficacy variables* and the *life quality variables* were not significantly associated with the outcome.

### Factors Predicting Anxiety Symptom Remission

Table 3 presents the results from univariate and multiple logistic regression analyses of variables predicting anxiety remission. In the univariate analysis, the only *primary illness variable* that was associated with anxiety remission, measured as BAI < 10, at 6 months was ‘Anxiety severity’ at baseline. Thus, a higher anxiety severity at baseline, measured by BAI, was associated with a lower remission rate at 6 months (OR = 0.45, 95% CI = 0.30,0.65, p < 0.001), i.e. one extra point in baseline BAI score reduced the odds of remission at 6-month BAI score by 55%. Out of the *comorbidity variables*, one was associated with anxiety remission at 6 months: A higher ‘Depression severity’ at baseline, measured by BDI-II, was associated with a lower remission rate at 6 months (OR = 0.67, 95% CI = 0.45,0.98, p = 0.043). Finally, the only *self-efficacy variable* that was associated with anxiety remission at 6 months was the self-efficacy measure ‘Self-efficacy to obtain help from community, family, friends’ from the SEMCD scale. Thus, a higher self-efficacy (to obtain help) at baseline was associated with a higher remission rate at 6 months (OR = 1.53, 95% CI = 1.09,2.20, p = 0.017).

**Table 3:** Predictors of anxiety remission at 6-month follow-up, univariate and multivariate logistic regression

	**Univariate**			**Multivariate**		
	**OR**	**95% CI**	**p-value**	**OR**	**95% CI**	**p-value**
**Demographic variables**						
**Age**	0.96	0.70,1.31	0.796			
**Sex**						
Male	0.74	0.36,1.47	0.392			
Female	1					
**Marital status**						
Married	0.97	0.46,1.99	0.943			
Not married	1					
**Educational level**						
Lower Secondary	1					
Upper Secondary	2.07	0.76,6.41	0.177			
Vocational or Bachelors’ degree	1.34	0.42,4.61	0.632			
Masters’ degree or Ph.D	2.60	0.86,8.74	0.103			
**Employment status**						
Employed	1					
Under education	0.65	0.27,1.46	0.307			
Unemployed	0.63	0.30,1.30	0.218			
**Primary illness variables**						
**Diagnosis (type of anxiety disorder)**						
Generalized anxiety disorder	1					
Panic disorder/ agoraphobia	1.55	0.74,3.32	0.252			
Social anxiety disorder/obsessive–compulsive disorder	0.99	0.38,2.51	0.979			
**Previous psychological/ medical treatment**						
No previous treatment	1					
Previous treatment	1.18	0.62,2.23	0.613			
**Anxiety severity (BAI**^**a**^**)**	0.45	0.30,0.65	** < 0.001**	0.54	0.32,0.89	**0.018**
**Comorbidity variables**						
**Comorbid depression severity (BDI-II**^**b**^**)**	0.67	0.45,0.98	**0.043**	0.79	0.46,1.35	0.396
**General psychological problems and symptoms of psychopathology (SCL-90-R**^**c**^**)**	0.68	0.53,1.04	0.085	1.00	0.54,1.84	0.999
**Personality disorder (SAPAS**^**d**^** > 2)**	0.68	0.34,1.32	0.262			
**Functional level variables**						
**Disability and functional impairment (SDS**^**e**^**)**	0.93	0.65,1.33	0.691			
**Life quality variables**						
**Well-being (WHO5**^**f**^**)**	0.89	0.61,1.28	0.533			
**Health-related quality of life (EQ-5D-3L**^**g**^**)**	1.50	0.99,2.40	0.072	1.32	0.85,2.18	0.236
**Self-efficacy variables**						
**Self-efficacy (personal control) (IPQ-R**^**h**^**)**	0.94	0.68,1.28	0.677			
**Self-efficacy (obtain help) (SEMCD**^**i**^**)**	1.53	1.09,2.20	**0.017**	1.44	0.99,2.14	0.062
**Self-efficacy (control/ manage symptoms) (SEMCD**^**i**^**)**	1.30	0.94,1.82	0.117			
**Intervention/treatment condition**						
Collabri Flex (CC)	1.82	0.96,3.57	0.075	1.43	1.02,2.03	**0.042**
Consultation liaison (CL)	1			1		

^a^BAI: Beck Anxiety Inventory

^b^BDI-II: Beck Depression Inventory

^c^SCL-90-R: Symptom Checklist

^d^SAPAS: Structured Assessment of Personality Abbreviated Scale

^e^SDS: Sheehan Disability Scale

^f^WHO5: WHO Well-Being Index

^g^EQ-5D-3L: European Quality of Life

^h^IPQ-R: Illness Perception Questionnaire Revised

^i^SEMCD: Self-Efficacy to Manage Chronic Disease Scales

In the multiple logistic regression analysis, only one variable remained significantly associated with anxiety remission at 6 months: A higher ‘Anxiety severity’ at baseline was associated with a lower remission rate at 6 months (OR = 0.54, 95% CI = 0.32,0.89, p = 0.018).

No association was found between the *demographic variables* representing socioeconomic status (i.e. marital status, educational level, and employment status), the *primary illness variable* ‘Diagnosis’ (type of anxiety disorder), or the *comorbidity variables* ‘General psychological problems and symptoms of psychopathology’ and ‘Personality disorder’, and the outcome. Furthermore, the remaining two *self-efficacy variables* and the *life quality variables* were not significantly associated with the outcome.

### Prediction From Machine-Learning Models

To identify non-linear predictors of the outcome, two machine-learning models were tested against an intercept-only ‘null’ linear regression model. Table 4 shows Pearson correlation coefficients for random forests (r = -0.068) and XGBoost (r = -0.148), respectively. The very low correlation coefficients show that there was no predictive performance of the machine-learning models.

**Table 4:** Pearson correlation coefficients of machine-learning model predictions versus the observed outcome

**Random forests**	**XG Boost**
-0,068	-0,148

### Control for Treatment Moderating Effects

To take into account that all participants in the RCT received an intervention (collaborative care or consultation liaison), we controlled for treatment moderating effects. Table S2 (Online Resource) shows the treatment moderating effects, by including interaction terms between significant predictor variables and the treatment condition variable in linear regression analysis. The treatment condition was only close to having a moderating effect on the association between one *primary illness variable* and anxiety symptom change at 6 months: A higher ‘Anxiety severity’ at baseline, measured by BAI, was associated with a higher symptom reduction at 6 months, and this association was stronger for patients receiving collaborative care compared to patients receiving consultation liaison (diff = -2.23, p = 0.058). Table S3 (Online Resource) shows that the treatment condition had no moderating effects on the associations between five independent variables and anxiety remission at 6 months, when including interaction terms in logistic regression analysis.

Furthermore, we implemented the causal forest machine-learning approach to identify potential baseline parameters with a predictive value for the benefit of the intervention, i.e. reduction in anxiety symptoms, measured by BAI, at 6 months for collaborative care versus consultation liaison. The relative importance of the variables is listed in Table S4 (Online Resource). We divided the variables into a low and a high effect of collaborative care versus consultation liaison, defined by a reduction in BAI, and examined the variables that led to a higher effect of the intervention, as shown in Table S5 (Online Resource). When the causal forest model predictions were applied to five training sets, there was no significant value from the model in predicting which patients had increased benefit from collaborative care, as shown in Table S6 (Online Resource).

## Discussion

In this study of 214 participants with anxiety disorders, we found that the *primary illness variable* ‘Anxiety severity’ at baseline was the strongest predictor of anxiety symptom reduction at 6 months. However, when using remission as the endpoint, a higher anxiety severity at baseline was associated with a lower chance of remission. Thus, the definition of the outcome variable determined the direction of the association. This difference could be due to the criteria for remission being easier to achieve if the baseline BAI score is relatively low. Conversely, if symptom improvement is defined as a large reduction in symptoms, this can be achieved without falling below the remission cut-off value when the baseline BAI score is high. The literature on the impact of anxiety severity at baseline on prognosis also shows contradictory results, which could also be explained by the use of different definitions of the outcome [13, 14, 17–19]. Other methodological issues of the literature include different anxiety disorders being studied.

Furthermore, the baseline *comorbidity variables* ‘General psychological problems and symptoms of psychopathology’ and ‘Depression severity’ were associated with a lower reduction in anxiety symptoms at 6 months. Thus, severe anxiety at baseline but without comorbidities predicted a greater reduction in anxiety symptoms at 6 months, whereas the presence of comorbidities reduced the chance of symptom reduction. However, comorbid depression was not retained as a significant predictor in the multiple regression models. This can be explained by the correlation coefficient of moderate strength between the baseline variables ‘Depression severity’ and ‘Anxiety severity’. The findings from the literature on the interactions between co-occurring anxiety and depression during treatment are conflicting [17–19, 44–46]. Some studies have found that comorbid depression adversely affects the treatment outcome of anxiety-specific treatments and increase the risk of treatment failure [13, 14]. Conversely, other studies have demonstrated that cognitive behavioral therapy and pharmacotherapy have a broad response and reduce both anxiety-specific symptoms and comorbid depression symptoms thus, overall comorbidity does not affect the treatment outcome for the anxiety disorder [17, 45]. However, a meta-analysis of the influence of comorbidity on the treatment outcome in different anxiety disorders found that comorbidity differentially impacts the outcome for specific anxiety diagnostic subgroups [17]. The relationship between overall comorbidity and treatment outcome for mixed anxiety was found to be negative, whereas there was a positive relationship between overall comorbidity and treatment outcome for panic disorder and/or agoraphobia, post-traumatic stress disorder, and obsessive–compulsive disorder, and no relationship with social anxiety disorder and specific phobias [17]. If the relationship between comorbidity and treatment outcome varies as a function of specific anxiety diagnostic subgroups, this could lead to personalization and better efficacy of treatment. Regarding a temporal relationship, one study of a collaborative care intervention suggests that anxiety reduction mediates the subsequent change in depression to a greater extent than vice versa [45]. Insight into the temporal course of change could also have great clinical value.

Moreover, among the *self-efficacy variables,* the measure ‘Self-efficacy to obtain help from community, family, friends’ from the SEMCD scale predicted a higher anxiety symptom reduction at 6 months. However, this association was no longer significant in multiple linear regression, but near significant in multiple logistic regression. An explanation for this could be that we measured self-efficacy separately by three sub-scales instead of using a multi-item measure, which might have affected the ability to capture the construct accurately. Potentially, the self-efficacy measure that we found had a predictive value, captures a person’s specific coping strategies more than a person’s general belief in one’s capabilities to successfully participate in treatment or an intervention. If so, our finding refers to a person’s ability to obtain help from family and friends, and indicate that having supportive social relations and networks has a positive impact on symptom reduction. Previous studies have consistently demonstrated that a greater perceived self-efficacy is associated with a better prognosis [13, 22], as self-efficacy enhances task motivation and affect the degree of effort put into completing the task [22].

Participating in the collaborative care intervention was associated with a higher symptom reduction and remission than participating in the consultation liaison intervention. This finding confirmed the overall result of the Collabri Flex study, favoring collaborative care [25]. When controlling for treatment moderating effects, the treatment condition (collaborative care versus consultation liaison) was only close to having a moderating effect on the association between anxiety baseline severity and symptom reduction at 6 months. This indicates that patients with a high anxiety severity at baseline benefit more from collaborative care than they would have from consultation liaison. Furthermore, the causal forest machine-learning model showed no significant value in predicting which patients had increased benefit from collaborative care. Thus, our results show that collaborative care had an effect on all patients regardless of patient characteristics related to demography, illness, comorbidity, functional level, life quality, and self-efficacy.

We found no associations between any of the *demographic variables,* including those representing socioeconomic status (i.e. educational level and employment status) or the *primary illness variable* ‘Anxiety diagnosis (diagnostic subgroup)’ respectively, and the 6-month outcome. The reason for not being able to measure a predictive value of socioeconomic status could be due to the setting of our RCT in primary care, whereas most other studies of predictors of anxiety remission are conducted in psychiatric settings [6, 10–12], where the sociodemographic composition of the patients is different [47]. In addition, despite socioeconomic status being a commonly reported predictor in the literature [10–13, 15, 48], studies vary in which parameters are used for measuring socioeconomic status thus, other studies also find no association [6, 49, 50]. In contrast to other studies that have found an association between different anxiety disorders and prognosis, we found no significant difference. This could be due to the relatively small sample size in our study thus, limiting the number of individuals in each diagnostic subgroup. Consequently, the statistical power to detect an effect of these variables was limited. We combined social anxiety disorder and /obsessive–compulsive disorder, and panic disorder/agoraphobia was overrepresented in the sample. This, in addition to the abovementioned power limitation, might explain why we did not detect anxiety disorder differences, as any associations might have been concealed by the combination of different disorders. Furthermore, in the overall RCT, 28.8% of the included patients with a primary anxiety diagnosis also presented with a secondary anxiety diagnosis [25]. As secondary anxiety diagnosis could not be taken into account in the present study, this could have further obscured the results. Finally, we found no association between *life quality variables* despite the well-established significance of life quality for remission throughout the literature [51, 52]*.*

### Strengths and Limitations

It was a strength of the present study that we were able to include multiple potential predictors, including characteristics related to baseline demography, illness, comorbidity, functional level, life quality, and self-efficacy. Likewise, we compared several anxiety diagnostic subgroups. Hence, the generalizability of the results of our study to a broad patient group is good. Furthermore, data was analyzed using a variety of statistical models and two different measures/definitions of the outcome variable were compared, all of which increased our trust in the results. Finally, the study was carried out in a primary care setting, where the vast majority of people with anxiety are treated [53].

Our findings should also be considered in the context of several limitations. First, a relatively small sample size limited the statistical power to detect any differences in variables with several categories, including educational level, employment status, and anxiety diagnostic subgroups. Secondly, some anxiety diagnostic subgroups were combined due to a low number of cases. Thus, some disorders were underrepresented, and we were not able to study agoraphobia and obsessive–compulsive disorder separately or post-traumatic stress disorder at all. Consequently, this could have concealed possible associations and limited the power of the analyses to detect differences. Thirdly, we did not test for all interactions between predictor variables, except for interactions with treatment condition. The predictors could be interconnected in their influence on symptom reduction and remission, and taking such interactions into account could have given a more realistic picture of the multicausal factors.

### Implications

There are several implications of our findings. Our results highlight the importance of managing both anxiety and comorbid psychiatric disorders, in particular comorbid depression, during treatment. This supports the existing literature on the beneficial use of broadly targeted interventions, especially since many patients in a real-world setting present with comorbid disorders [45]. Further research is needed on how comorbidities differentially impact the prognosis for different anxiety disorders. In addition, research should examine how anxiety disorders and comorbid disorders temporally change during treatment. Such research scopes could provide great clinical insights that could lead to personalization and better efficacy of treatment. In addition, an interpretation of our results might suggest that patients with low levels of self-efficacy could benefit from therapeutic strategies designed to strengthen and enhance their self-efficacy, thereby leading to an added treatment benefit and better prognosis. Finally, long-term research into predictors for recurrence of anxiety could identify specific characteristics of particularly vulnerable patients who need additional or more intensive treatment.

## Conclusion

Specific predictors for anxiety symptom reduction and remission at 6 months included baseline anxiety severity and psychiatric comorbidities. Our study contributes important prognostic information on baseline patient characteristics associated with 6-month symptom reduction and remission. This knowledge could be valuable for more personalized treatment decisions when managing anxiety disorders in primary care or when designing new interventions. Given the high prevalence of anxiety disorders and the substantial costs to the world economy, personalized treatment could improve treatment efficacy and hopefully limit treatment failure and the development of a fluctuating course or recurrence of anxiety disorders.

## Supplementary Information

Below is the link to the electronic supplementary material.

Supplementary file1 (PDF 341 KB)

## Data Availability

Any requests for data sharing should be directed to the corresponding author. Data that are shared upon reasonable request will be anonymized.
