# Supplementary material for: Predictors of Symptom Reduction and Remission Among People with Anxiety: Secondary Analyses from a Randomized Controlled Trial
Source: Psychiatr Q. 2024 Jul 18;95(3):447–67. doi: 10.1007/s11126-024-10081-y (PMC11420326; doi:10.1007/s11126-024-10081-y)
Supplement: Supplementary file 1 — Supplementary file1 (PDF 341 KB) [file 11126_2024_10081_MOESM1_ESM.pdf]

SUPPLEMENTARY MATERIAL (ONLINE RESOURCE)

Predictors of symptom reduction and remission among people with anxiety: secondary analyses from a randomized controlled trial

In Psychiatric Quarterly

Authors: Marte Ustrup, Thomas Christensen, Nadja Kehler Curth, Kimmie Heine, Anders Bo Bojesen, Lene Falgaard Eplov

Copenhagen Research Unit for Recovery, Mental Health Center Amager, Mental Health Services in the Capital Region of Denmark, Hans Bogbinders Allé 3, 2300, Copenhagen, Denmark

Corresponding author: Marte Ustrup, marte.ustrup@regionh.dk

Table S1. Correlation matrix

	Outcome: Anxiety severity (BAI[a])	Treatment condition	Age	Sex	Marital status	Personality disorder (SAPAS[b])	Anxiety severity baseline (BAI[a])	Comorbid dep. severity (BDI-II[c])	Diasability and function impairment (SDS[d])	Self-efficacy (personal control) (IPQ-R[e])	Self-efficacy (control/ manage symptom) (SEMCD[f]	Self-efficacy (obtain help) (SEMCD[f]	Well-being (WHO5[g])	General psychological problem/ psychopathology (SCL-90-R[h])	Health-related quality of life (EQ-5D-3L[i])	Lower 2nd education	Vocat. or Bachelors degree education	Upper 2nd education	Masters' degree or Ph.D. education	Under education	Unemployed	Employed	Diagnosis PD[j]/agoraphobia	Diagnosis SAD[k]/ OCD[l]	Diagnosis GAD[m]	No previous treatment	Previous treatment
**Outcome: Anxiety severity (BAI[a])**	1	-0,17	0,04	0,05	-0,04	0,1	-0,14	0,06	-0,05	0,03	-0,04	-0,17	0,12	0,04	0,04	0,1	0,03	-0,07	-0,03	-0,01	0,06	-0,04	-0,12	0,11	0,03	-0,09	0,09
**Treatment condition**	-0,05	1	-0,08	-0,09	-0,1	-0,05	0,03	0,06	0,11	0,13	0,04	-0,03	0,01	0,05	-0,05	0,01	0,02	-0,06	0,05	0,01	0,01	-0,02	0,12	-0,04	-0,1	0,01	-0,01
**Age**	-0,09	-0,11	1	0,16	0,44	-0,1	-0,12	-0,11	0,02	-0,13	0,08	0,03	0	-0,24	-0,03	-0,12	0,06	-0,09	0,16	-0,57	0,37	0,15	0,1	-0,24	0,11	-0,18	0,18
**Sex**	0,01	-0,09	0,17	1	-0,03	0,01	-0,23	-0,08	-0,07	0,01	0,02	-0,03	0,1	-0,13	0,07	0,1	-0,11	-0,01	0,05	-0,16	0,07	0,07	0,07	0,01	-0,09	0	0
**Marital status**	-0,05	-0,1	0,39	-0,03	1	0	0	-0,05	0,01	-0,03	0,13	0,15	0,02	-0,18	-0,06	-0,04	0,04	-0,08	0,09	-0,29	0,12	0,14	-0,02	-0,1	0,11	-0,05	0,05
**Personality disorder (SAPAS[b])**	0,1	-0,05	-0,11	0,01	0	1	-0,01	0,19	0,03	0,08	-0,16	-0,13	-0,06	0,19	-0,03	0,06	0,01	-0,04	-0,02	0,02	-0,09	0,07	-0,22	0,22	0,04	-0,05	0,05
**Anxiety severity baseline (BAI[a])**	-0,03	0,06	-0,14	-0,24	-0,02	0,02	1	0,47	0,51	-0,17	-0,24	-0,08	-0,49	0,63	-0,39	-0,02	-0,01	0,11	-0,11	0,13	-0,04	-0,08	0,13	0,06	-0,19	-0,05	0,05
**Comorbid dep. severity (BDI-II[c])**	0,12	0,05	-0,12	-0,1	-0,04	0,19	0,48	1	0,63	-0,12	-0,49	-0,35	-0,65	0,77	-0,39	0,1	-0,04	0,03	-0,08	0,12	0,03	-0,13	-0,04	0,13	-0,07	0,04	-0,04
**Diasability and function impairment (SDS[d])**	0,05	0,13	-0,07	-0,09	-0,01	0,02	0,52	0,63	1	-0,13	-0,29	-0,21	-0,6	0,63	-0,5	0	0,02	-0,06	0,05	-0,08	0,13	-0,06	0,09	0,11	-0,19	0,01	-0,01
**Self-efficacy (personal control) (IPQ-R[e])**	-0,04	0,13	-0,19	-0,02	-0,01	0,07	-0,14	-0,1	-0,08	1	0,34	0,24	0,19	-0,15	0,17	-0,08	0,12	-0,05	0,01	0,02	-0,15	0,12	-0,04	0,01	0,03	-0,01	0,01
**Self-efficacy (control/ manage symptoms) (SEMCD[f])**	-0,12	0,03	0,08	0,01	0,13	-0,16	-0,27	-0,5	-0,32	0,34	1	0,47	0,41	-0,43	0,24	-0,05	0,07	-0,02	0	-0,05	0,03	0,02	0,02	-0,06	0,03	-0,02	0,02
**Self-efficacy (obtain help) (SEMCD[f])**	-0,18	-0,03	0,01	-0,04	0,16	-0,15	-0,12	-0,34	-0,24	0,23	0,47	1	0,22	-0,36	0,14	-0,04	0,04	0,02	-0,04	-0,04	-0,04	0,07	0,09	-0,1	-0,01	0,06	-0,06
**Well-being (WHO5[g])**	-0,02	0	0,06	0,11	0,05	-0,05	-0,5	-0,67	-0,6	0,16	0,43	0,23	1	-0,63	0,39	-0,01	-0,08	0,05	0,04	-0,01	-0,05	0,06	-0,07	-0,08	0,14	-0,06	0,06
**General psychological problems/ psychopathology (SCL-90-R[h])**	0,11	0,05	-0,24	-0,13	-0,19	0,19	0,65	0,75	0,64	-0,12	-0,46	-0,35	-0,64	1	-0,43	0,08	-0,04	0,06	-0,1	0,19	-0,06	-0,11	0,09	0,13	-0,22	0,02	-0,02
**Health-related quality of life (EQ-5D-3L[i])**	-0,02	0	-0,03	0,04	-0,04	-0,04	-0,41	-0,43	-0,5	0,15	0,28	0,14	0,43	-0,44	1	-0,18	0,02	-0,01	0,16	0,01	-0,2	0,18	-0,15	0,09	0,08	-0,02	0,02
**Lower 2nd education**	-0,03	0,01	-0,05	0,1	-0,04	0,06	-0,02	0,09	0	-0,12	-0,06	-0,05	-0,02	0,1	-0,17	1	-0,22	-0,38	-0,2	0,06	0,08	-0,13	0,09	0,07	-0,16	0,17	-0,17
**Vocat. or Bachelors' degree education**	-0,04	0,02	0	-0,11	0,04	0,01	-0,05	-0,04	0,03	0,12	0,07	0,07	-0,1	-0,04	0,04	-0,22	1	-0,47	-0,25	0,08	-0,04	-0,03	-0,07	0,04	0,04	-0,05	0,05
**Upper 2nd education**	0,08	-0,06	-0,03	-0,01	-0,08	-0,04	0,12	0,03	-0,06	-0,05	-0,02	0,02	0,06	0,05	-0,02	-0,38	-0,47	1	-0,44	0,11	0	-0,1	0,09	-0,06	-0,05	0,06	-0,06
**Masters' degree or Ph.D. education**	-0,02	0,05	0,08	0,05	0,09	-0,02	-0,08	-0,08	0,05	0,05	0,01	-0,05	0,05	-0,11	0,14	-0,2	-0,25	-0,44	1	-0,28	-0,03	0,27	-0,12	-0,02	0,15	-0,17	0,17
**Under education**	0,07	0,01	-0,49	-0,16	-0,29	0,02	0,15	0,11	-0,03	0,02	-0,06	-0,02	-0,04	0,21	0,02	0,06	0,08	0,11	-0,28	1	-0,4	-0,5	-0,19	0,23	0,01	0,12	-0,12
**Unemployed**	-0,03	0,01	0,41	0,07	0,12	-0,09	-0,08	0,01	0,09	-0,16	0,03	-0,06	-0,04	-0,07	-0,21	0,08	-0,04	0	-0,03	-0,4	1	-0,6	0,12	-0,09	-0,05	-0,14	0,14

**Employed**	-0,03	-0,02	0,04	0,07	0,14	0,07	-0,05	-0,11	-0,06	0,13	0,02	0,07	0,07	-0,11	0,18	-0,13	-0,03	-0,1	0,27	-0,5	-0,6	1	0,05	-0,11	0,04	0,02	-0,02
**Diagnosis: PD[j]/ agoraphobia**	-0,11	0,12	0,12	0,07	-0,02	-0,22	0,12	-0,03	0,07	-0,05	0,04	0,08	-0,06	0,09	-0,15	0,09	-0,07	0,09	-0,12	-0,19	0,12	0,05	1	-0,51	-0,63	0,03	-0,03
**Diagnosis: SAD[k]/ OCD[l]**	0,06	-0,04	-0,24	0,01	-0,1	0,22	0,06	0,12	0,13	0,01	-0,08	-0,09	-0,08	0,13	0,08	0,07	0,04	-0,06	-0,02	0,23	-0,09	-0,11	-0,51	1	-0,34	0,12	-0,12
**Diagnosis: GAD[m]**	0,06	-0,1	0,08	-0,09	0,11	0,04	-0,18	-0,08	-0,19	0,04	0,02	0	0,13	-0,22	0,09	-0,16	0,04	-0,05	0,15	0,01	-0,05	0,04	-0,63	-0,34	1	-0,14	0,14
**No previous treatment**	-0,05	0,01	-0,14	0	-0,05	-0,05	-0,05	0,05	0,03	-0,02	-0,01	0,08	-0,06	0,02	-0,03	0,17	-0,05	0,06	-0,17	0,12	-0,14	0,02	0,03	0,12	-0,14	1	-1
**Previous treatment**	0,05	-0,01	0,14	0	0,05	0,05	0,05	-0,05	-0,03	0,02	0,01	-0,08	0,06	-0,02	0,03	-0,17	0,05	-0,06	0,17	-0,12	0,14	-0,02	-0,03	-0,12	0,14	-1	1

Numbers listed on the left side under the diagonal shows Pearson correlation coefficients. Numbers listed on the right side above the diagonal shows Spearman's rank correlation coefficients.

[a] BAI: Beck Anxiety Inventory. [b] SAPAS: Structured Assessment of Personality Abbreviated Scale. [c] BDI II: Beck Depression Inventory. [d] SDS: Sheehan Disability Scale. [e] IPQ-R: Illness Perception Questionnaire Revised. [f] SEMCD: Self-Efficacy to Manage Chronic Disease Scales. [g] WHO5: WHO Well-being Index. [h] SCL-90-R: Symptom Checklist. [i] EQ-5D-3L: European Quality of Life. [j] PD: Panic disorder. [k] SAD: Social anxiety disorder. [l] OCD: Obsessive compulsive disorder. [m] GAD: Generalized anxiety disorder.

Table S2. Predictors associated with benefit from collaborative care as opposed to consultation liaison, linear regression

	Consultation liaison (CL)		Collaborative care (CC)		Difference	
	β	**p-value**	***β***	**p-value**	**Diff *β***	**Diff. p-value**
**Primary illness variables**						
**Anxiety severity (BAI[a])**	-3.29	<0.001	-5.52	<0.001	-2.23	**0.058**
**Comorbidity variables**						
**Comorbid depression severity (BDI-II[b])**	-1.63	0.052	-0.55	0.587	-1.08	0.412
**General psychological problems and symptoms of psychopathology (SCL-90-R[c])**	-1.63	0.045	-1.31	0.186	0.32	0.804
**Life quality variables**						
**Health-related quality of life (EQ-5D-3L[d])**	2.40	0.005	1.91	0.050	-0.48	0.710
**Self-efficacy variables**						
**Self-efficacy (obtain help) (SEMCD[e])**	-0.58	0.484	-1.05	0.290	-0.47	0.718

Estimates are stratified by intervention/treatment condition. The difference of each predictor's strength in the two groups is tested as an interaction term between treatment condition and predictor variable.

[a] BAI: Beck Anxiety Inventory. [b] BDI-II: Beck Depression Inventory. [c] SCL-90-R: Symptom Checklist.

[d] EQ-5D-3L: European Quality of Life. [e] SEMCD: Self-Efficacy to Manage Chronic Disease Scales.

Table S3. Predictors associated with benefit from collaborative care as opposed to consultation liaison, logistic regression

	Consultation liaison (CL)		Collaborative care (CC)		Difference	
	OR	p-value	OR	p-value	Diff. OR	Diff. p-value
**Primary illness variables**						
**Anxiety severity (BAI[a])**	0.39	0.002	0.48	0.003	1.25	0.575
**Comorbidity variables**						
**Comorbid depression severity (BDI-II[b])**	0.43	0.004	0.54	0.007	1.25	0.549
**General psychological problems and symptoms of psychopathology (SCL-90- R[c])**	0.55	0.023	0.41	0.001	0.74	0.420
**Life quality variables**						
**Health-related quality of life (EQ-5D-3L[d])**	1.98	0.040	1.99	0.021	1.01	0.988
**Self-efficacy variables**						
**Self-efficacy (obtain help) (SEMCD[e])**	1.40	0.170	1.83	0.012	1.31	0.426

Estimates are stratified by intervention/treatment condition. The difference of each predictor's strength in the two groups is tested as an interaction term between treatment condition and predictor variable.

[a] BAI: Beck Anxiety Inventory. [b] BDI-II: Beck Depression Inventory. [c] SCL-90-R: Symptom Checklist.

[d] EQ-5D-3L: European Quality of Life. [e] SEMCD: Self-Efficacy to Manage Chronic Disease Scales.

Table S4. Variables ranked by the relative importance for the outcome, from the causal forest machine-learning method

	Variable importance
**Age**	0.075
**General psychological problems and symptoms of psychopathology (SCL-90-R[a])**	0.067
**Comorbid depression severity (BDI-II[b])**	0.067
**Anxiety severity (BAI[c])**	0.065
**Self-efficacy (control/ manage symptoms) (SEMCD[d])**	0.064
**Well-being (WHO5[e])**	0.064
**Disability and functional impairment (SDS[f])**	0.051
**Self-efficacy (personal control) (IPQ-R[g])**	0.050
**Health-related quality of life (EQ-5D-3L[h])**	0.047
**Diagnosis: Generalized anxiety disorder**	0.043
**Previous treatment: No previous psychological/medical treatment**	0.042
**Self-efficacy (obtain help) (SEMCD[d])**	0.041
**Employment: Employed**	0.034
**Previous treatment: Previous psychological/medical treatment**	0.033
**Employment: In education**	0.032
**Diagnosis: Panic anxiety/agoraphobia**	0.032
**Sex**	0.026
**Education: Upper secondary**	0.026
**Marital status**	0.022
**Personality disorder (SAPAS[i] > 2)**	
**Education: Vocational or bachelor's degree**	0.019
**Employment: Unemployed**	0.014
**Diagnosis: Social anxiety disorder/obsessive compulsive disorder**	0.009
**Education: Master or PhD**	0.008
**Education: Lower secondary**	0.001
**Treatment**	0

[a] SCL-90-R: Symptom Checklist. [b] BDI II: Beck Depression Inventory. [c] BAI: Beck Anxiety Inventory. [d] SEMCD: Self-Efficacy to Manage Chronic Disease Scales. [e] WHO5: WHO Well-being Index. [f] SDS: Sheehan Disability Scale. [g] IPQ-R: Illness Perception Questionnaire Revised. [h] EQ-5D-3L: European Quality of Life. [i] SAPAS: Structured Assessment of Personality Abbreviated Scale.

Table S5. Baseline characteristics of predicted high vs. low effect groups, from the causal forest machine-learning method

	**Low effect** *N = 102*		**High effect** *N = 112*		
	N	**(%)**	***N***	**(%)**	**p-value**
**Demographic variables**					
	**Mean**	**SD**	**Mean**	**SD**	**p-value**
**Age**	34.52	14.43	38.58	14.64	**0.017**
	N	**(%)**	***N***	**(%)**	**p-value**
**Sex**					
Female	78	76.5	72	63.4	
Male	24	23.5	40	35.7	0.052
**Marital status**					
Unmarried	85	83.3	74	66.1	
Married	17	16.7	38	33.9	**0.004**
**Educational level**					
Lower Secondary	18	17.6	14	12.5	
Upper Secondary	42	41.2	54	48.2	
Vocational or Bachelors' degree	25	24.5	20	17.9	
Master or PhD	17	16.7	24	21.4	**0.035**
**Employment status**					
Employed	36	35.3	56	50.0	
Under education	24	23.5	29	25.9	
Unemployed	42	41.2	27	24.1	**0.022**
**Primary illness variables**					
**Diagnosis**					
Generalized anxiety disorder	29	28.4	34	30.4	
Panic disorder/agoraphobia	51	50.0	54	48.2	
Social anxiety disorder/ obsessive-compulsive disorder	22	21.6	24	21.4	0.95
**Previous psychological/medical treatment**					
No previous treatment	71	69.6	40	35.7	
Previous treatment	31	30.4	72	64.3	**<0.001**
	**Mean**	**SD**	**Mean**	**SD**	**p-value**
**Anxiety severity (BAI[a])**	22.56	5.87	28.07	9.98	**<0.001**
**Comorbidity variables**					
**Comorbid depression severity (BDI-II[b])**	23.37	8.30	20.34	10.42	**0.012**
**General psychological problems and symptoms of psychopathology (SCL-90-R[c])**	112.57	41.91	108.52	49.50	0.516

	N	(%)	*N*	(%)	p-value
**Personality disorder (SAPAS[d] > 2)**	40	39.2	39	34.8	0.601
**Functional level variables**	**Mean**	**SD**	**Mean**	**SD**	**p-value**
**Disability and functional impairment (SDS[e])**	16.54	6.19	15.58	7.92	0.573
**Life quality variables**					
**Well-being (WHO5[f])**	34.24	14.71	39.46	20.75	0.057
**Health-related quality of life (EQ-5D-3L[g])**	0.69	0.15	0.67	0.19	0.618
**Self-efficacy variables**					
**Self-efficacy (personal control) (IPQ-R[h])**	21.20	4.11	20.59	4.09	0.124
**Self-efficacy (obtain help) (SEMCD[i])**	6.17	1.84	6.65	2.08	**0.027**
**Self-efficacy (control/manage symptoms) (SEMCD[i])**	5.33	1.78	6.13	1.84	**0.001**

[a] BAI: Beck Anxiety Inventory. [b] BDI-II: Beck Depression Inventory. [c] SCL-90-R: Symptom Checklist. [d] SAPAS: Structured Assessment of Personality Abbreviated Scale. [e] SDS: Sheehan Disability Scale. [f] WHO5: WHO Well-being Index. [g] EQ-5D-3L: European Quality of Life. [h] IPQ-R: Illness Perception Questionnaire Revised. [i] SEMCD: Self-Efficacy to Manage Chronic Disease Scales.

Table S6. Change from baseline for predicted high vs. low effect groups, from the causal forest machine-learning method

Causal Forest	Treatment	Mean	95% CI	N (test set)	p-value
High effect	0	-8.77	-11.35,-6.19	52	
Low effect	0	-6.05	-8.33,-3.76	43	0.123
High effect	1	-11.00	-14.16,-7.84	55	
Low effect	1	-10.52	-12.72,-8.31	64	0.798
Correlation with full-sample prediction: 0.36					
